# State of mission: Battery management with neural networks and electrochemical AI

**DOI:** 10.1016/j.isci.2025.113593

**Published:** 2025-10-07

**Authors:** Cengiz S. Ozkan, Mihrimah Ozkan

**Affiliations:** 1 Materials Science and Engineering, University of California, Riverside, Riverside, CA, USA; 2 Department of Chemistry, University of California, Riverside, Riverside, CA, USA; 3 Department of Mechanical Engineering, University of California, Riverside, Riverside, CA, USA; 4 Department of Electrical Engineering, University of California, Riverside, Riverside, CA, USA

**Keywords:** Applied sciences, Energy management, Energy Systems

## Abstract

This work introduces a hybrid modeling framework for advanced battery management that combines neural ordinary differential equations (Neural ODEs) with physics-informed neural networks (PINNs) to achieve physically consistent, data-driven predictions of battery behavior. Sequential learning models, including long short-term memory (LSTMs) and Transformers, are integrated to capture temporal dependencies and provide continuous-time, high-fidelity estimation. A central contribution is the introduction of the state of mission (SOM), a mission-aware diagnostic metric that quantifies whether a battery can successfully complete a specific operational task. Unlike conventional measures such as state of charge (SOC) or state of health (SOH), SOM integrates internal state evolution, mission profiles, and safety constraints to forecast mission feasibility. The framework was validated through simulations and experimental data from the NASA PCoE and Oxford datasets. Results demonstrate robust prediction of coupled electrochemical-thermal dynamics and mission outcomes, offering a forward-looking tool for next-generation battery management systems in electric vehicles, aerial systems, and grid storage.

## Highlights

• Neural ODE framework models electrochemical, thermal, and aging dynamics
• Physics-informed learning ensures physically consistent battery state predictions
• SOM enables mission-aware battery state estimation for real-time decision-making
• Hybrid AI model supports predictive, safe, and adaptive battery management systems

## Graphical abstract

**Figure:**
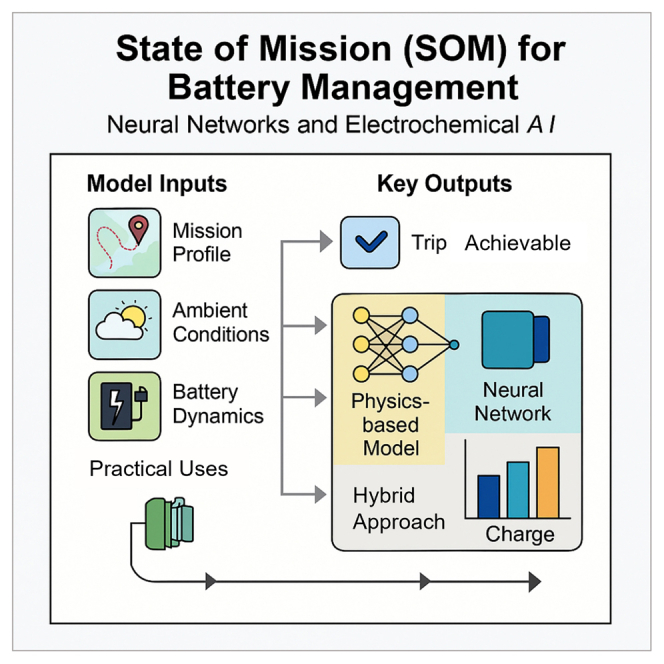

## Introduction

The rapid expansion of battery-dependent technologies, particularly in demanding sectors such as electric vehicles (EVs) and large-scale grid energy storage, has led to a corresponding increase in the complexity and performance expectations placed upon these energy storage systems.^1^^,^^2^^,^^3^ Central to the effective operation of these systems is the battery management system (BMS), a critical suite of electronics and software responsible for overseeing and controlling the battery pack.^4^^,^^5^^,^^6^ The BMS plays an indispensable role in ensuring the safety,^7^ operational efficiency,^8^ reliability,^9^ and extended lifespan of the battery.^1^^,^^10^ Its core functionalities encompass continuous monitoring of battery parameters, accurate estimation of internal states, sophisticated thermal management to maintain optimal operating temperatures, and robust fault diagnosis to prevent hazardous conditions. A fundamental prerequisite for the successful execution of these BMS functions is the accurate and real-time estimation of various battery states, including but not limited to the state of charge (SOC),^11^^,^^12^ state of health (SOH),^13^ state of energy (SOE),^14^ state of power (SOP),^15^ state of temperature (SOT),^16^ and state of safety (SOS).^17^ The precision of these estimations directly dictates the BMS’s ability to optimize battery performance and ensure its safe operation throughout its service life.

The escalating energy and power densities of modern batteries, while enabling advancements in application performance, concurrently bring batteries closer to their operational and safety thresholds.^1^ This proximity to the limits elevates the risk of undesirable phenomena such as thermal runaway, which can lead to catastrophic failures, and accelerated degradation, which shortens the battery’s useful life.^18^ Consequently, the ability to accurately estimate and predict internal states—for instance, the internal temperature distribution across the cell or pack^19^ and the progression of various degradation mechanisms^20^—becomes paramount for proactive and preventative management. This pressing need for enhanced predictive capabilities is a primary driver for the development of more advanced and comprehensive battery state estimation models capable of capturing these intricate, coupled dynamics.

Historically, battery modeling has relied heavily on two dominant paradigms: traditional physics-based models and purely data-driven approaches. Physics-based models, such as equivalent circuit models (ECMs)^21^ and electrochemical models like the Doyle-Fuller-Newman (DFN)^22^ or single particle model (SPM), offer strong interpretability and grounding in electrochemical principles. While ECMs are computationally efficient and suitable for estimating basic parameters such as SOC, they often fail to capture non-linear behaviors and degradation under dynamic conditions.^12^ More detailed electrochemical models provide higher fidelity by describing ion transport and reaction kinetics but are computationally intensive and require extensive parameterization, with many parameters difficult to obtain or adapt over time.^23^^,^^24^^,^^25^ This complexity renders them unsuitable for real-time use in typical BMS hardware.

Despite their respective strengths, both physics-based and data-driven modeling approaches face notable limitations when applied independently. Physics-based models, while grounded in electrochemical theory and offering interpretability, often suffer from high computational cost, limited adaptability, and reliance on hard-to-obtain parameters—making them less suitable for real-time battery management. In contrast, data-driven models can efficiently learn complex patterns from operational data but frequently lack physical consistency, interpretability, and generalizability beyond the training domain. These complementary shortcomings underscore the need for a unified modeling paradigm that retains the physical rigor of mechanistic models while leveraging the flexibility and learning capacity of modern machine learning techniques. This motivates the development of hybrid frameworks that integrate both perspectives to enable robust, scalable, and real-time battery state estimation under diverse operating conditions.

Purely data-driven models—ranging from classical machine learning algorithms to deep neural networks—can learn complex input-output relationships directly from operational data without relying on explicit physical formulations.^23^ However, they are often criticized for their “black-box” nature, limited interpretability, and difficulty in extrapolating beyond the training domain.^26^ Their effectiveness heavily depends on large, diverse, and high-quality datasets, and they may yield physically inconsistent results under unseen or noisy conditions.^26^^,^^27^ These challenges have fueled growing interest in hybrid modeling approaches that integrate physics-based insights with data-driven flexibility. Such frameworks seek to capitalize on the strengths of both paradigms while mitigating their weaknesses, offering a promising path toward high-fidelity, interpretable, and scalable battery management solutions for next-generation energy systems.^28^^,^^29^

**Figure 1:**
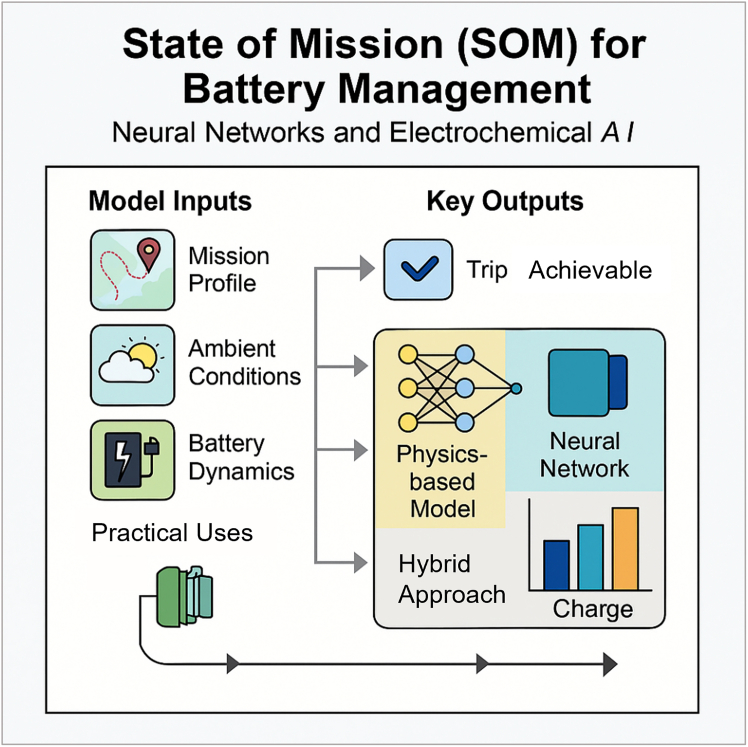
SOM for battery management This illustration depicts the proposed hybrid framework that integrates mission-specific inputs with a physics-informed Neural ODE architecture to assess battery readiness in real time. Model inputs—including mission profile, ambient conditions, and battery dynamics—are processed through a hybrid learning pipeline combining a neural network with a physics-based model. The framework enables the computation of the battery state of mission (SOM), a mission-aware state function that forecasts whether a battery can complete a specific task. By embedding electrochemical, thermal, and degradation dynamics, this system enhances the accuracy and interpretability of state estimation. The output supports real-time decisions on trip feasibility and charge sufficiency, offering transformative capabilities for advanced BMSs in applications such as EVs, autonomous drones, and grid-scale energy storage.

The primary objective of this paper is to introduce a developed comprehensive mathematical and computational framework based on continuous-time neural networks (neural ordinary differential equations [Neural ODEs]) for modeling the dynamic evolution of battery states (Figure 1). This framework uniquely integrates electrochemical, thermal, and degradation sub-models within a unified architecture, ensuring both physical fidelity and predictive accuracy. Leveraging physics-informed neural networks (PINNs) for adherence to physical laws and advanced sequential architectures (long short-term memory [LSTMs]/Transformers) for temporal data processing, the model enables robust and interpretable battery state estimation. The proposed framework integrates three complementary modeling paradigms—Neural ODEs, PINNs, and sequential learning architectures—to form a cohesive, end-to-end battery state estimation system. Specifically, historical time-series data such as voltage, current, and temperature are processed by a sequential model (e.g., LSTM or Transformer) to estimate the battery’s initial internal state vector, which is often unobservable but critical for accurate forecasting. This estimated state serves as the initial condition for a Neural ODE, which simulates the continuous-time evolution of electrochemical, thermal, and degradation states. To ensure physical consistency, the Neural ODE is trained with embedded constraints derived from fundamental governing equations (e.g., mass and charge conservation, reaction kinetics), implemented using PINN principles. The resulting framework combines temporal awareness, physical interpretability, and dynamic generalization—enabling mission-aware predictions that are both data driven and physics constrained. A key innovation introduced in this work is the state of mission (SOM)—a mission-aware battery state function that forecasts whether the battery can successfully complete a specific operational task (Figure 1). By incorporating mission context into state estimation, the framework advances BMSs from reactive monitoring to proactive, goal-oriented decision-making.

## Results

### Defining and interpreting internal states in BMSs

To enable advanced battery management strategies, a comprehensive understanding of key internal battery states—commonly referred to as SOX (state of X)—is essential. These states include metrics such as SOC, SOH, SOE, and others that collectively define the battery’s performance, safety, and remaining lifespan. Each state offers unique insights into battery behavior and operational conditions, and together, they form the backbone of effective diagnostics, control, and decision-making in BMSs. As energy storage systems become more complex—particularly in EVs and grid applications—the demand for real-time, accurate estimation of these internal states has grown significantly. Misestimating these values can lead to performance degradation, safety hazards, or premature battery failure.

Table 1 provides a detailed summary of the principal SOX variables, including their definitions, physical significance, and the factors influencing their evolution.^30^^,^^31^^,^^32^ This tabular overview captures how various internal and external conditions—such as temperature, aging, charge/discharge rate, and system load—interact to influence these battery states. The intent is to equip researchers and engineers with a holistic reference for integrating state estimation models into hybrid physics-AI battery frameworks. By systematically organizing this information, the table serves as both a conceptual foundation and a practical guide for developing more reliable and responsive BMS algorithms.

**Table 1:** Summary of key battery internal states (SOX): Definitions, significance, and influencing factors

Battery state	Definition	Physical significance	Influencing factors
State of charge (SOC)	current available charge relative to nominal/max capacity	indicates remaining usable energy; prevents overcharge/discharge	current rate, temperature, SOH, self-discharge
State of health (SOH)	measure of current battery condition relative to new state	crucial for predicting lifespan and scheduling maintenance	cycle count, age, DOD, charge/discharge rate, temp history
State of energy (SOE)	ratio of remaining energy to max available energy, accounting for voltage	accounts for voltage impact on usable energy; more accurate than SOC	SOC, voltage, temperature, load, SOH
State of power (SOP)	maximum peak power deliverable or absorbable under current conditions	essential for dynamic applications like EV acceleration or braking	SOC, SOH (resistance), temp, voltage/current limits
State of temperature (SOT)	temperature distribution across battery cell/pack	impacts safety, performance, and aging; critical for thermal management	ambient temp, current, internal resistance, dissipation
State of resistance (SOR)	internal opposition to current flow, including ohmic and polarization resistance	affects voltage drop, heating, power loss, and performance degradation	SOH, SOC, temperature, current, AC frequency
State of functionality (SOF)	ability to perform a specific task given current overall state	evaluates real-time functionality and task readiness	application-specific loads, SOC, SOH, SOP, SOT
State of availability (SOA)/SoAP	available power considering all safe operating constraints	guides safe and effective power usage based on operating conditions	SOC, SOH, voltage, temperature, current limits
State of safety (SOS)	assessment of battery safety status considering risk conditions	supports early fault detection to prevent catastrophic failure	voltage, temperature, current, degradation, mechanical abuse
State of life (SOL)/RUL	predicted remaining time/cycles before reaching end-of-life	supports life cycle planning and end-of-life forecasting	cycles, temperature, usage patterns, degradation mechanisms

### Continuous-time neural networks for modeling battery dynamics

An emerging and powerful approach within the hybrid modeling paradigm is the use of Neural ODEs. Unlike traditional deep learning models that rely on discrete layers to transform inputs, Neural ODEs define the evolution of a system’s state as a continuous-time process. Specifically, they use a neural network to parameterize the derivative function:

$$\frac{dX}{dt}=f\left(X,t,\theta \right)\text{,}$$

where X is the system state, t is time, and θ represents the neural network parameters.^26^

The resulting state trajectory X(t) is obtained by integrating this differential equation using numerical ODE solvers. This formulation aligns naturally with physical systems like batteries, where the evolution of internal states is inherently continuous.

Given that battery behavior unfolds through complex, time-dependent processes—such as ion diffusion, electrochemical reactions, heat transfer, and material degradation—Neural ODEs offer a highly flexible framework for modeling these dynamics without imposing rigid structural assumptions.^33^ Rather than prescribing a fixed mathematical structure (e.g., an equivalent circuit or reduced-order electrochemical model), Neural ODEs can learn the underlying vector field that governs the battery’s internal state evolution directly from data.^34^ This makes them especially attractive for capturing multi-scale, non-linear, and coupled processes that are otherwise difficult to model explicitly. Table 2 summarizes the key advantages of Neural ODEs in the context of dynamic battery modeling.

**Table 2:** Key advantages of Neural ODEs for modeling dynamic battery systems^35^

Advantage	Description
Continuous-time modeling	naturally handles continuous-time processes, aligning with the inherent continuous evolution of battery internal states
Irregularly sampled data	effectively manages time-series data with non-uniform sampling intervals, which is typical in real-world BMS data logging
Adaptive computation	utilizes numerical ODE solvers with adaptive step sizes, enabling more efficient computation than fixed-step models like RNNs, especially for smooth dynamics
Memory efficiency	supports constant memory cost during backpropagation (for reversible solvers), unlike RNNs whose memory requirements grow with model depth or sequence length^26^

While Neural ODEs provide a powerful framework for learning the dynamic behavior of battery systems, their inherent black-box nature poses challenges in interpretability and consistency with physical laws.^36^ To address this, PINNs offer a compelling solution by embedding domain knowledge directly into the learning process.^37^ PINNs incorporate known physical laws—typically formulated as partial or ordinary differential equations (PDEs/ODEs), conservation laws, or boundary and initial conditions—into the neural network training by augmenting the loss function with residuals from these equations.^27^ In doing so, the network is not only trained to minimize prediction errors against observed data but also to adhere to established physical constraints. In the context of battery modeling, this allows Neural ODEs to be guided by principles of electrochemistry, thermodynamics, and degradation kinetics, resulting in models that are not only more accurate but also more generalizable, especially when data are limited. This hybridization enhances physical plausibility and supports robust extrapolation to unseen operating scenarios.^38^

To further improve the ability to model temporal battery behavior, particularly when processing time-series data such as current, voltage, and temperature histories, advanced sequential architectures can be integrated with Neural ODEs. Architectures such as LSTM networks and Transformers are particularly well suited for encoding input sequences or refining the output of Neural ODE solvers. Their ability to capture long-range dependencies and learn temporal patterns makes them valuable tools in augmenting the predictive capabilities of physics-aware neural models for real-world battery management applications.

### Proposed integrated Neural ODE framework for battery state evolution

This section details a proposed framework that synergistically combines Neural ODEs with electrochemical, thermal, and degradation sub-models,^39^ informed by PINN principles and integrated with sequential deep learning architectures (LSTMs/Transformers) for robust battery state evolution modeling.

Table 3 systematically itemizes the critical internal variables that define a battery’s electrochemical, thermal, and degradation status. By defining the state vector X with these components, the Neural ODE can learn their coupled evolution. The governing principles listed guide the formulation of physics-informed constraints within the PINN framework, ensuring that the learned dynamics adhere to fundamental battery science. This structured definition is crucial for the scope and complexity of the Neural ODE, the selection of appropriate physical equations for PINN loss terms, and the interpretation of learned dynamics and serves as a foundational reference.

**Table 3:** Key battery state variables for dynamic modeling^40^^,^^41^^,^^42^^,^^43^

State variable	Symbol	Governing principle/equation type	Relevance to Neural ODE
Li-ion concentration in solid electrode (anode)	cs,n (r,x,t)	Fick’s law of diffusion (PDE)	component of state vector X; its spatial distribution or key averages (surface, bulk) are tracked
Li-ion concentration in solid electrode (cathode)	cs,p (r,x,t)	Fick’s law of diffusion (PDE)	component of state vector X; its spatial distribution or key averages (surface, bulk) are tracked
Li-ion concentration in electrolyte	Ce (x,t)	Nernst-Planck equation/Fick’s law (PDE)	component of state vector X; its spatial distribution or key averages are tracked
Solid phase potential (anode)	ϕs,n (x,t)	Ohm’s law/charge conservation (PDE/algebraic)	component of state vector X or auxiliary variable calculated from X
Solid phase potential (cathode)	ϕs,p(x,t)	Ohm’s law/charge conservation (PDE/algebraic)	component of state vector X or auxiliary variable calculated from X
Electrolyte phase potential	ϕe(x,t)	Ohm’s law/charge conservation (PDE/algebraic)	component of state vector X or auxiliary variable calculated from X
Interfacial current density (anode)	jn(x,t)	Butler-Volmer equation (algebraic, depends on cs, ce, ϕs, ϕe,T)	calculated within f(X,t,θ) to drive changes in cs,ce
Interfacial current density (cathode)	jp(x,t)	Butler-Volmer equation (algebraic, depends on cs,ce,ϕs,ϕe,T)	calculated within f(X,t,θ) to drive changes in cs,ce
Temperature	T(x,t)	energy balance/heat equation (PDE)	component of state vector X; its spatial distribution or key averages (core, surface) are tracked
SEI layer thickness (anode)	LSEI (x,t)	reaction kinetics/diffusion (ODE/PDE)	component of state vector X; evolves based on T,ϕs,ϕe
Fraction of active material (anode)	ϵactive,n (x,t)	empirical/mechanistic degradation law (ODE)	component of state vector X; evolves based on stress, cycling, T
Fraction of active material (cathode)	ϵactive,p (x,t)	empirical/mechanistic degradation law (ODE)	component of state vector X; evolves based on stress, cycling, T
Concentration/amount of plated lithium (anode)	cLi,plated (x,t)	side reaction kinetics (Butler-Volmer/Tafel) (ODE/PDE)	component of state vector X; evolves based on T,ϕs,ϕe, ce
Total capacity loss due to SEI	Qloss, SEIt)	integral of SEI formation current (ODE)	component of state vector X or derived from LSEI
Total capacity loss due to LAM	Qloss,LAM(t)	integral of LAM rate (ODE)	component of state vector X or derived from ϵactive

### Detailed architecture: Coupling Neural ODEs with electrochemical, thermal, and degradation sub-models

The proposed framework centers around a core Neural ODE that models the continuous-time evolution of a comprehensive battery state vector. This core is augmented by physical constraints and interfaced with sequential models for input/output processing.

Central Neural ODE core: the heart of the system is a Neural ODE^33^^,^^44^ described by the following equation:

$$\frac{dX\left(t\right)}{dt}=f_{NN}(X\left(t\right),t,I\left(t\right),T_{amb}\left(t\right),\theta_{NN}\text{,}$$

where X(t) is the high-dimensional battery state vector (defined in Table 1), I(t) is the applied current, T_amb_(t) is the ambient temperature, and f_NN_ is a neural network (parameterized by θ_NN_) that learns the complex, coupled dynamics of the state variables. The function f_NN_ is not necessarily a single monolithic neural network; it can be a composite function where different neural network components model specific physical processes (e.g., heat generation rate and reaction kinetics for side reactions), and these components are combined according to known physical structures (e.g., within the energy balance equation or degradation rate laws). This “gray-box” approach allows for injecting more physical knowledge into the structure of f_NN_ itself, potentially making the learning task more tractable and the model more interpretable.

State vector X(t): as detailed in Table 3, X(t) includes variables representing the following.

○ Electrochemical states: key Li-ion concentrations (e.g., cs,n,surf, cs,p,surf, ce,avg) and potentials or overpotentials.
○ Thermal states: cell temperatures (e.g., T_core_, T_surf_, or T_avg_).
○ Degradation states: solid electrolyte interphase (SEI) layer thickness (LSEI), lost Li inventory (QLi_loss), active material fractions (ϵactive,n/p), and amount of plated Li (Qplated_Li).

Physics-informed components (enforced via PINN loss terms).

○ Electrochemical module: the evolution of electrochemical states within X(t) will be constrained by residuals from DFN/SPM equations. For example, the rate of change of surface concentrations dcs,surf/dt will be linked to flux jrxn (from Butler-Volmer) and bulk diffusion. The Neural ODE might learn corrections or unmodeled dependencies for these terms.
○ Thermal module: the temperature state(s) T in X(t) will evolve according to an energy balance equation. For instance, dTavg/dt will be constrained by ρcp dtdTavg = Qgen_NN(X,I)−Qloss(T_avg_,T_amb_), where Qgen_NN is a neural network component within fNN learning the heat generation rate (itself possibly constrained by Bernardi-like terms or Butler-Volmer overpotentials), and Qloss represents heat dissipation (e.g., convection).
○ Degradation module: the evolution of degradation states like dLSEI/dt, dϵactive/dt, and dQplated_Li/dt within X(t) will be constrained by their respective kinetic equations. For example,
  ▪ dLSEI/dt constrained by models like kSEI (T,ϕs, …)/LSEI.^45^
  ▪ dϵactive/dt constrained by LAM models dependent on stress factors or cycle rate.^46^
  ▪ dQplated_Li/dt constrained by plating current density jplating from Butler-Volmer kinetics.^47^ The neural network components within fNN can learn the complex dependencies of these rate constants and driving forces on the overall state X(t) and operational inputs.

LSTM/Transformer integration.

○ Input encoder: an LSTM or Transformer module processes historical sequences of measured voltage Vhist, current Ihist, and temperature Thist to estimate the initial state vector X(0) for the Neural ODE. The accuracy of X(0) is crucial, as ODE solutions are highly sensitive to initial conditions. This encoder learns the mapping from observable history to the unobservable internal states.
○ Output decoder (optional but recommended for specific tasks): another LSTM or Transformer module can take the state trajectory X(t) (or selected components of it) output by the Neural ODE to predict specific future observable quantities (e.g., future V_term_(t+Δt)) or to compute user-relevant metrics like SOC, SOH, or RUL.

### Input variables, state variables, and output predictions

A clear definition of the model’s inputs, internal states, and outputs is fundamental for its specification and implementation. Table 4 provides a clear specification for the inputs, internal states, and outputs of the integrated Neural ODE system. It distinguishes between inputs for initial state estimation, dynamic inputs to the Neural ODE, the evolving state vector components, and the final outputs relevant for BMS applications. This structured I/O definition is crucial for designing data pipelines, understanding information flow, defining loss terms, and communicating the model’s capabilities.

**Table 4:** Input/output specification for the integrated neural ODE model

Category	Parameter name	Symbol	Description	Data type/units
Input to system (for X(0) estimation)	historical current profile	Ihist(t′)	time series of applied current prior to t0	sequence of floats (A)
	historical voltage profile	Vhist(t′)	time series of measured terminal voltage prior to t0	sequence of floats (V)
	historical temperature profile	Thist(t′)	time series of measured (e.g., surface) temperature prior to t0	sequence of floats (°C or K)
Neural ODE initial state input	initial state vector	X(0)	vector of all initial electrochemical, thermal, and degradation states (output of input encoder); see Table 1 for components	vector of floats (various units)
Neural ODE dynamic input	applied current at time t	I(t)	instantaneous applied current during the ODE integration period	float (A)
	ambient temperature at time t	Tamb(t)	instantaneous ambient temperature during the ODE integration period	float (°C or K)
Internal state vector component	Avg. Li-ion conc. in anode surface	cs,n,surf(t)	average lithium concentration at the surface of anode active material particles	float (mol/m^3^)
	Avg. Li-ion conc. in cathode surface	cs,p,surf(t)	average lithium concentration at the surface of cathode active material particles	float (mol/m^3^)
	Avg. Li-ion conc. in electrolyte	ce,avg(t)	average lithium concentration in the electrolyte	float (mol/m^3^)
	average cell temperature	Tavg(t)	average temperature of the battery cell	float (°C or K)
	SEI layer thickness (anode)	LSEI(t)	average thickness of the SEI layer on the anode	float (m)
	lost lithium inventory	QLi_loss(t)	cumulative amount of lithium lost to side reactions	float (Ah or Coulombs)
	active material fraction (anode)	ϵactive,n(t)	fraction of anode material still electrochemically active	float (dimensionless, 0–1)
	active material fraction (cathode)	ϵactive,p(t)	fraction of cathode material still electrochemically active	float (dimensionless, 0–1)
	(*other states from*Table 1*as needed*)	…	…	…
System output (examples)	predicted terminal voltage	Vpred(t)	predicted terminal voltage of the battery (can be a direct state or calculated from X(t) via an output function/decoder)	float (V)
	predicted average/Surface temperature	Tpred(t)	predicted average or surface temperature of the battery(component of X(t) or decoded)	float (°C or K)
	estimated state of charge	SOCest(t)	estimated SOC, derived from components of X(t) (e.g., cs,n,avg, QLi_loss, ϵactive,n)	float (%, 0–100)
	estimated state of health (capacity)	SOHC,est(t)	estimated SOH based on capacity, derived from X(t) (e.g., QLi_loss, ϵactive,n/p)	float (%, 0–100)
	estimated etate of health (resistance)	SOHR,est(t)	estimated SOH based on internal resistance, derived from X(t) (e.g., LSEI, Tavg, ϵactive,n/p)	float (%, 0–100)
	estimated state of energy	SOEest(t)	estimated SOE, derived from SOCest(t), Vpred(t), and SOH	float (Wh or kWh, or %)
	estimated state of power	SOPest(t)	estimated SOP, calculated based on X(t) and operational limits	float (W)

### Implementation overview of the Neural ODE framework

This section outlines the conceptual and architectural implementation of the proposed Neural ODE framework for dynamic battery modeling. A multi-level flowchart helps visualize the training loop and system components, while an overview of the Python code structure provides insights into modular organization and computational flow. At the highest level (level 1), the system receives time-series battery operation data (e.g., voltage, current, and ambient temperature) and, through an initial state estimator—such as an LSTM or Transformer encoder—generates the estimated initial battery state vector X(0). This estimate is then passed into the core component of the model: a Neural ODE defined by the differential equation dX/dt = f_NN_(X,t,I(t),T_amb_(t),θ), where f_NN_ is a neural network that learns the coupled battery dynamics. The resulting state trajectory X_pred_(t) may be fed into a decoder network (if needed) to generate predictions for observable outputs like terminal voltage and temperature.

Loss computation is a key part of training. It integrates three elements: (1) data loss quantifying the mismatch between predicted and observed outputs (e.g., voltage), (2) physics-based residuals from governing equations (electrochemical, thermal, and degradation), and (3) constraints enforcing boundary and initial conditions. These components together form the total loss L_total_, which is minimized via an optimizer such as Adam. Notably, PINN principles are incorporated by embedding physical equations into the loss function, thereby ensuring that the learned dynamics remain consistent with battery physics. During inference, the trained model outputs predictions for both observable parameters and internal battery states like SOC and SOH.

At a more detailed level (level 2), the structure of the neural function f_NN_ is elaborated. Rather than a monolithic network, f_NN_ comprises modular components that model electrochemical processes (e.g., reaction kinetics), thermal behavior (e.g., heat generation and dissipation), and degradation mechanisms (e.g., SEI growth and lithium plating). These modules compute intermediate quantities, which are then combined to produce the derivative vector dX/dt governing the evolution of the state vector. Matrix operations and non-linear activations drive the computations in these components, and where applicable, spatial derivatives are incorporated using discretized operators or autograd techniques.

To implement this architecture, a modular Python codebase is recommended. Key modules include data_loader.py (for data preprocessing and batching), sequential_models.py (defining encoders and decoders), and neural_ode_f.py (defining the dynamics function f_NN_). Additional files such as physics_constraints.py implement PINN-based loss terms, solve_ode.py wraps the ODE solver, and training_pipeline.py orchestrates the full training loop. Evaluation and configuration are handled by evaluation.py and config.py, respectively. The framework relies heavily on PyTorch and the torchdiffeq package for differentiable ODE solving, enabling end-to-end training of the entire system with physics-based constraints and temporal awareness.

The entire training pipeline’s reliance on the ability to backpropagate gradients through the ODE solver underscores the criticality of libraries like torchdiffeq. The adjoint sensitivity method, typically implemented in such libraries, allows for memory-efficient computation of these gradients. Furthermore, handling spatial PDEs within the PINN loss requires careful consideration. If the state vector X includes spatially discretized quantities (e.g., ce(xi,t) for multiple spatial points xi), then computing terms like ∇·(De∇ce) requires evaluating spatial derivatives. This can be achieved using automatic differentiation if the neural network itself outputs the spatial profile as a function of spatial coordinates or by applying finite difference stencils to the components of Xpred(t) that represent the discretized field. The choice impacts the structure of fNN and the physics_constraints module.

### SOM for batteries

While standard SOX states (SOC, SOH, SOP, etc.) provide crucial diagnostic information about a battery’s current intrinsic properties, they do not directly quantify the battery’s fitness to perform a specific, upcoming operational task. To address this, we propose a concept of “SOM” for batteries, drawing an analogy from readiness assessments in other critical systems.

#### Defining battery SOM

In many real-world applications—such as EVs, grid energy storage systems, and mission-critical operations—knowing the battery’s current status (like SOC) is often insufficient. What is truly needed is the ability to predict whether the battery can successfully complete a specific future operational profile. For instance, an EV driver needs assurance that the battery can support an entire planned trip, not just an update on current charge levels. Similarly, a grid operator must know whether a battery energy storage system can reliably provide contracted services such as frequency regulation for the coming hour.

This concept parallels established practices in the military and aerospace sectors, where the term SOM or “mission capability” denotes a system’s readiness to perform assigned tasks. In those domains, SOM assessments consider the health of subsystems, available resources, and environmental conditions to determine whether a platform—such as an aircraft or a spacecraft—is capable of fulfilling its mission. These are integrated, predictive evaluations that go beyond mere diagnostics.

Translating this idea to battery systems, we propose defining the battery SOM as a quantitative or probabilistic measure of a battery’s ability to successfully execute a predefined upcoming operational mission—be it a specific drive cycle, a grid service profile, or a flight plan for an electric aircraft. This assessment must account for safety margins, performance targets, and degradation thresholds, offering a forward-looking and task-specific perspective. In doing so, battery SOM shifts the paradigm from passive state estimation to active mission assurance.

#### Factors influencing battery SOM

The assessment of a battery’s SOM relies on a combination of internal conditions, predictive modeling, mission-specific demands, and operational constraints. First and foremost, the current battery state plays a critical role and is derived from the Neural ODE framework’s state vector X(t_0_). This vector encapsulates key metrics such as the initial SOC and SOE, which indicate the battery’s available energy; the SOH, including capacity and internal resistance, which affects energy efficiency and power limits; the SOP, reflecting the battery’s instantaneous power delivery capability; the SOT, which influences both performance and safety; and the SOS, indicating any pre-existing safety concerns or flags.

In addition to the initial state, the predicted state evolution—i.e., the trajectory of X(t) over the mission duration—provides insight into how these internal states will evolve under mission-specific conditions. This prediction is generated by the Neural ODE model and forms the backbone of forward-looking SOM assessment.

The mission requirements themselves are equally important and include the total duration of the mission (t_mission_), the time-varying power or current demand profile (P_demand_(t) or I_demand_(t)), the total energy required for successful mission completion (E_mission_), and the ambient environmental conditions expected throughout the mission (T_amb, mission_(t)).

Lastly, operational and safety constraints must be rigorously applied. These include a minimum allowable SOC at the end of the mission (SOC_min,end_), voltage limits (V_max_ and V_min_), maximum permissible cell or pack temperatures (T_max_), and constraints on charge/discharge rates (C-rates). Additionally, mission-specific limits on degradation—such as a cap on lithium inventory loss (ΔQ_Li,loss_)—must be considered to ensure long-term battery viability and warranty compliance. Together, these factors form a comprehensive basis for determining whether a battery can safely and reliably complete its intended mission.

#### Estimating battery SOM using the Neural ODE framework

The proposed Neural ODE framework offers a powerful and flexible approach for estimating a battery’s SOM, thanks to its capacity to model the complex, multi-physics evolution of battery states over time. The estimation process begins by defining the mission profile, which includes specifying the required input current or power profile I_mission_(t) or P_mission_(t), along with the ambient temperature trajectory T_amb, mission_(t), for the entire mission duration t∈[t_0_,t_0_+t_mission_].

Next, the initial state vector X(t_0_) is estimated using an input encoder—such as an LSTM network or a Transformer model—trained on recent historical battery data. This vector encompasses key internal states like SOC, SOH, SOT, and others, serving as the starting point for mission simulation.

The third step involves simulating the mission using the Neural ODE, defined as dX/dt = f_NN_ (X, t, I_mission_(t), T_amb, mission_(t), θ). The integration of this ODE from X(t_0_) across the mission duration yields the predicted state trajectory X_pred, mission_(t).

Once the trajectory is obtained, the system evaluates performance and safety constraints throughout the mission. This includes verifying that predicted voltage V_pred_(t) remains within the allowable range [V_min_, V_max_], that the predicted temperature T_pred_(t) does not exceed T_max_, and that the final predicted SOC at the end of the mission SOC_pred_(t_0_+t_mission_) is greater than or equal to the required minimum SOC_min,end_. Additional checks include ensuring C-rate compliance and acceptable degradation levels (e.g., lithium inventory loss).

Finally, the SOM is quantified. This can take multiple forms.

• A binary SOM classification (1 = mission feasible, 0 = not feasible), based on whether all constraints are satisfied.
• A probabilistic SOM, where the mission feasibility is expressed as a probability, particularly if uncertainty quantification is included in the Neural ODE framework—for example, through Bayesian neural networks or ensemble methods.
• A quantitative SOM score, reflecting mission feasibility on a continuous scale (e.g., 0%–100%), derived from safety margins, remaining energy, or deviations from optimal performance. This could be computed using expressions such as the following equation:

$$SOM=\min\left(\frac{{SOC}_{target,end}-{SOC}_{\min,end}}{{SOC}_{end}-{SOC}_{\min,end}},\frac{T_{\max}-T_{optimal}}{T_{\max}-T_{peak}},\ldots \right)\text{.}$$

**Figure 2:**
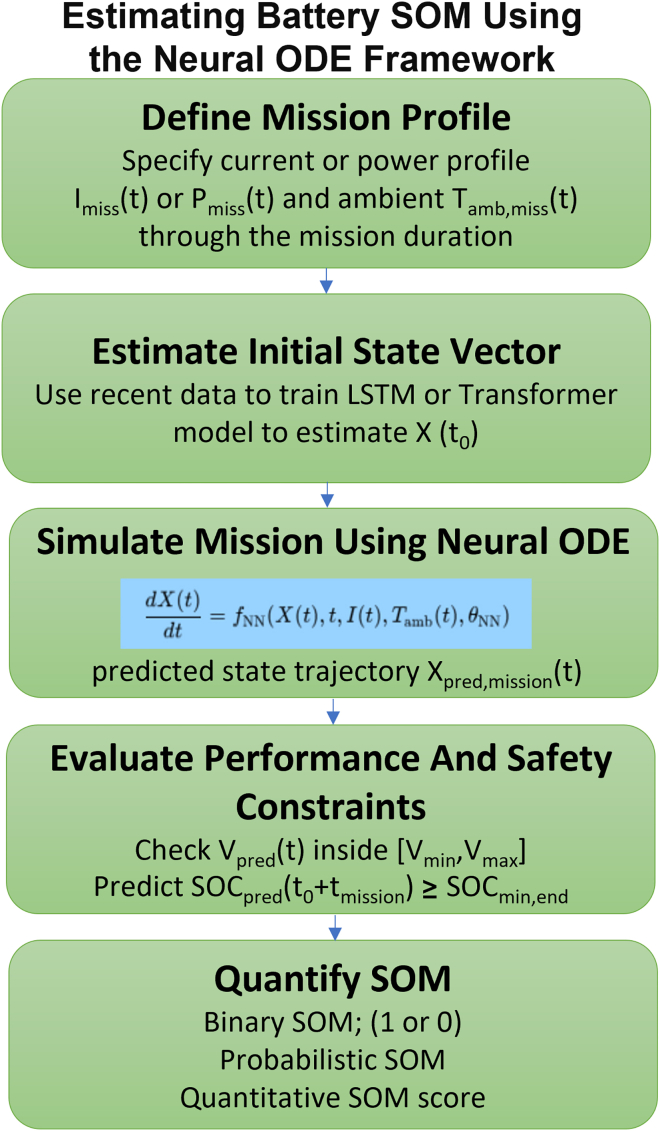
Flowchart for estimating battery SOM using the Neural ODE framework This flowchart illustrates the sequential process for estimating a battery’s state of mission (SOM) through a physics-informed Neural ODE framework. The procedure begins with the definition of a mission profile, including the required current or power input, I_mission_(t) or P_mission_(t),and the ambient temperature trajectory T_amb, mission_(t) over the mission duration. Next, the initial state vector X(t_0_) is estimated using a sequential input encoder such as an LSTM or Transformer, trained on historical data. In the third step, the mission is simulated using a Neural ODE, which integrates the dynamic state evolution dX/dt = f_NN_ (X, t, I_mission_(t), T_amb, mission_(t), θ) over time to yield the predicted state trajectory Xpred, mission(t)X_^7^}(t)Xpred, mission(t). The framework then evaluates whether the predicted voltage V_pred_(t) remains within specified safety bounds [Vmin, Vmax], the predicted temperature stays below a maximum threshold, and the final state of charge SOC_pred_(t_0_+t_mission_) exceeds a required minimum SOC_min,end_. Finally, the SOM is quantified as a binary classification (feasible/not feasible), a probabilistic score, or a continuous SOM value, providing actionable insights for mission planning and battery-aware decision-making.

This approach (Figure 2) enables a robust, forward-looking evaluation of mission readiness, providing battery-aware intelligence critical for high-reliability applications.

### Case study

In this work, we present two representative case studies (Table 5) to evaluate the generality and adaptability of the proposed SOM framework. The first examines a short-distance urban EV scenario, characterized by stop-and-go driving, moderate ambient temperatures, and relatively low power demand. The second focuses on a long-haul electric freight vehicle operating along a multi-segment route with varying terrain, elevated thermal stress, and sustained high load conditions. Together, these case studies demonstrate the SOM framework’s ability to dynamically adapt to diverse mission profiles—ranging from routine urban commutes to complex, high-demand logistics routes. The results confirm SOM’s potential as a general-purpose, mission-aware diagnostic and forecasting tool for intelligent battery management across a wide range of electric mobility and energy storage applications.

**Table 5:** Two case study comparison summary: Urban trip and long-haul multi-segment trip

Feature/metric	Urban EV (short trip)	Long-haul freight EV (multi-segment)
Route type	flat urban commute	mixed terrain with mountainous ascent
Distance	23 km	385 km
Segments	1	4 (urban, highway, mountainous, descent)
Ambient temperature range	18°C–32°C	26°C–42°C
Initial SOC	58%	87%
Initial SOH	∼87%	78%
Maximum C-rate	0.6C	1.2C
Thermal peak (core temp)	38.5°C	49.8°C
End-of-mission SOC	19.6%	16.3%
Lithium plating risk	none detected	near threshold (3.1%)
Degradation indicators	minimal	moderate SEI growth, increased SOR
Quantitative SOM score	92.4%	73.5%
SOM feasibility classification	feasible	feasible (low margin)
Recommended BMS action	none	thermal pre-conditioning, route control

#### Case study 1: SOM estimation for urban EV deployment

To demonstrate the practical applicability of the proposed SOM framework, we conducted a scenario-based analysis simulating a representative use case in urban electric mobility. This case study evaluates the ability of a lithium-ion battery pack in a passenger EV to complete a predefined city commute under realistic operational conditions using the physics-informed Neural ODE framework.

#### Mission profile and environmental context

**Figure 3:**
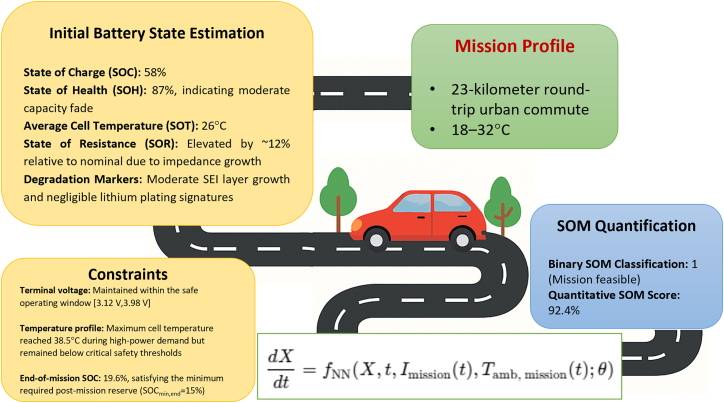
SOM estimation for a short-range urban EV scenario The mission involves a 23-kilometer round-trip urban commute under ambient temperatures ranging from 18°C to 32°C. The initial battery state is characterized by a moderate SOC (58%), good SOH (87%), slightly elevated internal resistance, and moderate degradation markers including SEI growth. Constraints for voltage, temperature, and post-mission SOC were all satisfied, with a maximum core temperature of 38.5°C and an end-of-mission SOC of 19.6%. The SOM framework, implemented via a Neural ODE model, confirmed mission feasibility with a binary SOM classification of 1 and a high quantitative SOM score of 92.4%, indicating strong margin and operational readiness under realistic urban driving conditions.

The mission selected for SOM evaluation corresponds to a 23-kilometer round-trip urban commute (Figure 3). The driving pattern emulates a typical metropolitan route incorporating a mix of stop-and-go traffic, moderate inclines, and brief highway segments. The ambient temperature was defined as a time-varying function over the mission duration, ranging from 18°C in the early morning to 32°C during mid-day, reflecting realistic environmental variability. The current demand profile, *I*_mission_(*t*), was constructed using a high-fidelity EV propulsion model aligned with standard drive cycles (e.g., WLTP Urban and Artemis Urban), incorporating transient loads, regenerative braking events, and realistic acceleration bursts.

#### Initial internal state estimation

Prior to mission execution, the initial battery internal state vector *X*(*t*_0_) was estimated using a Transformer-based sequential encoder trained on historical current, voltage, and temperature time-series data. The extracted initial conditions are as follows.

• SOC: 58%.
• SOH: 87%, indicating moderate capacity fade.
• Average cell temperature (SOT): 26°C.
• State of resistance: elevated by ∼12% relative to nominal due to impedance growth.
• Degradation markers: moderate SEI layer growth and negligible lithium plating signatures.

This initial state serves as the input to the Neural ODE solver, which models the evolution of internal battery states under mission-driven conditions.

#### Neural ODE-based mission simulation

The predicted evolution of the state vector over the mission duration was obtained by integrating the following Neural ODE:

$$\frac{dX}{dt}=f_{NN}\left(X\left(t\right),t,I_{mission}\left(t\right),T_{amb,mission}\left(t\right),\theta \right)\text{,}$$

where f_NN_ is a hybrid neural network informed by electrochemical, thermal, and degradation dynamics. The ODE integration spanned a mission duration of 55 min, capturing transient phenomena at sub-second resolution.

Key simulation outcomes are as follows.

• Terminal voltage: maintained within the safe operating window (3.12 V, 3.98 V).
• Temperature profile: maximum cell temperature reached 38.5°C during high-power demand but remained below critical safety thresholds.
• End-of-mission SOC: 19.6%, satisfying the minimum required post-mission reserve (SOC_min,end_ = 15%).
• Safety markers: no indication of thermal, electrical, or electrochemical limit violations throughout the mission trajectory.

While the initial SOC was 58% and the SOH remained relatively high at 87%, these traditional metrics alone would offer limited guidance as to whether the mission could be completed under real-world conditions. The SOM framework, by contrast, integrates these static values with mission-specific energy demands, environmental factors, and internal degradation indicators—offering a forward-looking assessment that confirms operational readiness with greater confidence and clarity.

#### Quantitative SOM evaluation

Based on the satisfaction of all predefined mission and safety constraints, the SOM was computed across three formulations.

• Binary SOM classification: 1 (mission feasible).
• Quantitative SOM score: 92.4%.
• Probabilistic SOM (if uncertainty modeling is included): not computed for this case but framework-compatible.

The high SOM score indicates strong mission feasibility given the current state and external conditions. Sensitivity analysis showed that reducing initial SOC below 55% or encountering ambient temperatures exceeding 40°C could reduce the SOM score below 70%, suggesting the need for pre-conditioning or mission replanning.

#### Comparison with traditional metrics

In contrast to conventional state metrics such as SOC or SOH, which offer static, decontextualized assessments, the SOM framework delivers a task-specific, forward-looking readiness evaluation. While an SOC of 58% might appear sufficient in isolation, the SOM metric provides a nuanced answer to the question: “Can this battery safely and reliably complete the upcoming mission?”—a critical insight for intelligent energy management in real-world EV deployments. This case study underscores the practical utility of SOM in dynamic, mission-oriented BMS design. By integrating predictive battery modeling with contextual mission data, the SOM framework facilitates proactive control strategies such as dynamic charging recommendations, real-time rerouting, and mission abort logic. As illustrated, SOM augments the functional depth of traditional battery diagnostics by bridging internal state estimation with operational intent—representing a critical step toward the realization of adaptive and intelligent BMS architectures.

### Advanced case study 2: SOM evaluation for long-haul electric freight operations

**Figure 4:**
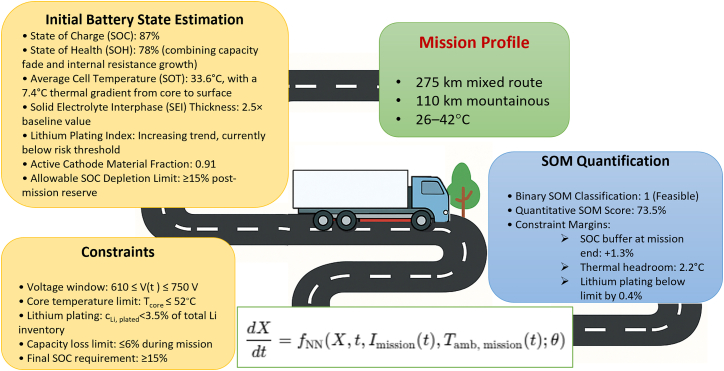
SOM estimation framework applied to a long-haul EV case study The mission profile comprises a 275 km mixed-terrain route, including 110 km of mountainous driving under ambient temperatures ranging from 26°C to 42°C. The initial internal battery state is characterized by a high SOC (87%), moderate SOH degradation (78%), elevated thermal gradients, and early-stage lithium plating and SEI formation. A Neural ODE model simulates the dynamic battery state evolution under mission-specific current and temperature profiles, constrained by voltage, thermal, and degradation thresholds. SOM quantification indicates mission feasibility with a quantitative SOM score of 73.5%, supported by sufficient SOC and thermal margins. This figure illustrates the multi-dimensional integration of physics-informed AI and mission-driven battery management.

To further demonstrate the robustness and decision-making capabilities of the proposed SOM framework, we present an advanced case study (Figure 4) involving a long-haul electric freight vehicle operating under demanding mission conditions. This scenario highlights the system’s ability to perform predictive battery state forecasting while accounting for multi-segment driving, variable environmental conditions, high C-rate demands, and degradation-aware safety constraints.

#### Mission profile and segment-specific load characteristics

The operational mission involves a 385-km delivery route executed by a class 8 electric freight truck equipped with a 700 V, 400 kWh lithium-ion battery pack. The route is subdivided into four distinct segments with varying terrain, payload conditions, and ambient temperatures. Table 6 summarizes the mission structure.

**Table 6:** Multi-segment route profile for long-haul freight mission

Segment	Distance (km)	Terrain type	Payload (%)	Ambient temperature	Peak C-rate
1	65	urban (stop-and-go)	100	26°C–30°C	0.5C
2	140	highway (steady speed)	100	30°C–35°C	0.7C
3	110	mountainous (climbing)	80	35°C–42°C	1.2C
4	70	suburban (descending)	60	33°C–28°C	0.4C

The power demand profile P_mission_(t) was synthesized using a high-fidelity vehicle dynamics model incorporating road grade, aerodynamic drag, regenerative braking, and rolling resistance.

#### Initial battery state estimation

The initial internal state vector X(t_0_) was estimated using a Transformer-based sequential encoder trained on 48 h of historical driving data. The encoded battery states are as follows.

• SOC: 87%.
• SOH: 78% (combining capacity fade and internal resistance growth).
• Average cell temperature (SOT): 33.6°C, with a 7.4°C thermal gradient from the core to the surface.
• SEI thickness: 2.5× baseline value.
• Lithium plating index: increasing trend, currently below risk threshold.
• Active cathode material fraction: 0.91.
• Allowable SOC depletion limit: ≥15% post-mission reserve.

These initial values set the boundary conditions for the subsequent simulation of the mission trajectory.

#### SOM estimation via Neural ODE simulation

The SOM trajectory was computed using the proposed physics-informed Neural ODE framework:

$$\frac{dX}{dt}=f_{NN}\left(X\left(t\right),t,I_{mission}\left(t\right),T_{amb,mission}\left(t\right),\theta \right)\text{,}$$

where f_NN_ is a hybrid neural network informed by electrochemical, thermal, and degradation dynamics. The ODE integration spanned a mission duration of 55 min, capturing transient phenomena at sub-second resolution.

Subject to the following constraints.

• Voltage window: 610 ≤ V(t) ≤ 750 V.
• Core temperature limit: T_core_ ≤52°C.
• Lithium plating: c_Li, plated_ <3.5% of total Li inventory.
• Capacity loss limit: ≤6% during mission.
• Final SOC requirement: ≥15%.

The simulation produced the following key outputs.

• Peak core temperature: 49.8°C during segment 3 (climbing under high load and ambient heat).
• Final SOC: 16.3%.
• Cumulative lithium plating: 3.1%.
• Voltage range during mission: 620–735 V.
• Degradation markers: within thresholds, but accelerating during final segment.

Although the battery began the mission with a high SOC of 87%, the SOM analysis revealed only a modest margin of feasibility (73.5%), primarily due to elevated thermal loads and emerging degradation effects not captured by SOC or SOH alone. This contrast underscores SOM’s advantage in synthesizing multiple internal and external variables to deliver a comprehensive mission-readiness forecast, beyond what static metrics can provide.

#### SOM quantification and interpretation

Based on the evaluation of internal dynamics and constraint adherence, the SOM was quantified as follows.

• Binary SOM classification: 1 (feasible).
• Quantitative SOM score: 73.5%.
• Constraint margins:
  ○ SOC buffer at mission end: +1.3%.
  ○ Thermal headroom: 2.2°C.
  ○ Lithium plating below limit by 0.4%.

Although the SOM indicates mission feasibility, the margin is modest, with most thresholds approached during segment 3. This suggests a mission-sensitive risk zone, particularly under hotter ambient conditions or unexpected surges in demand.

#### Implications for BMS strategy and decision-making

This case study reveals the value of SOM in enabling predictive and constraint-aware battery management. The BMS, informed by SOM analysis, can implement the following strategies.

• Thermal preconditioning: initiate active cooling prior to entering high-load segments.
• Adaptive driving recommendations: reduce acceleration in segment 3 to manage thermal rise.
• Charge planning for return trip: schedule a recharge or extended cool-down phase after mission completion.
• Degradation monitoring: log and compare SEI and lithium plating rates across repeated missions for long-term optimization.

In contrast to traditional state variables (e.g., SOC or SOH), SOM provides an integrated, mission-specific readiness forecast that includes electrochemical, thermal, and degradation-aware dimensions. Even though SOC was relatively high at mission start (87%), the SOM revealed that the system was only marginally prepared for the full profile due to thermal and aging constraints.

This advanced case study underscores the predictive strength and operational relevance of the SOM framework in complex, high-stakes battery applications. By enabling real-time mission feasibility analysis under multi-physics constraints, SOM supports a generation of adaptive BMSs—capable of transforming state estimation from a passive diagnostic tool to a proactive control function aligned with operational goals.

### Sensitivity analysis of SOM to initial SOC and ambient temperature

To assess the robustness of the SOM framework under variable initial conditions, we conducted a sensitivity analysis by simulating the same long-haul freight mission across a matrix of initial SOC levels and ambient temperatures. The results, summarized in Table 7, reveal a clear degradation in SOM scores with decreasing initial SOC and increasing ambient thermal stress. At favorable ambient conditions (25°C), the SOM remains above 90% even with initial SOC as low as 75%, indicating high mission feasibility. However, at elevated ambient temperatures (e.g., 42°C), the SOM drops below 60% when the initial SOC falls below 60%, signaling increased risk due to thermal limitations and reduced energy headroom. These results highlight the nonlinear and coupled influence of environmental and battery state variables on mission viability—an insight that static SOC- or SOH-based indicators fail to capture. Importantly, this analysis demonstrates the value of SOM in proactively identifying marginal mission conditions and informing adaptive strategies such as pre-mission charging or thermal pre-conditioning.

**Table 7:** Sensitivity of SOM to varying initial SOC at 3 ambient temperature levels

Initial SOC (%)	SOM @ 25°C	SOM @ 35°C	SOM @ 42°C
90%	97%	94%	88%
75%	91%	84%	73%
60%	77%	65%	52%

### Future directions: Incorporating uncertainty into SOM estimation

While the current framework produces deterministic estimates of the SOM, many real-world applications—particularly in safety-critical domains such as autonomous vehicles, aerial systems, and grid operations—require probabilistic forecasts that explicitly quantify uncertainty. Recognizing this need, future extensions of the SOM framework will incorporate uncertainty quantification through the following methodologies.

#### Bayesian neural ODEs

A Bayesian treatment of the Neural ODE model enables posterior distributions over the neural network parameters, offering principled estimation of epistemic uncertainty. Techniques such as Hamiltonian Monte Carlo or variational inference can be employed to approximate these distributions, allowing the model to express confidence bounds over predicted internal states and SOM outcomes.

#### Ensemble-based methods

Training an ensemble of Neural ODE models with different random initializations or data subsets provides an empirical distribution of predictions. The resulting spread in SOM outputs can be used to construct probabilistic feasibility intervals or confidence scores (e.g., standard deviation, percentiles).

#### Monte Carlo dropout

As a lightweight alternative, dropout layers can be retained during inference and sampled multiple times to simulate posterior draws. This approach has been shown to effectively capture uncertainty in deep learning models with minimal computational overhead.

#### Aleatoric uncertainty estimation

Noise-aware loss functions (e.g., heteroscedastic regression) can be incorporated to explicitly model measurement noise and environmental variability, offering more realistic forecasts under stochastic mission profiles.

#### Quantile SOM forecasting

Instead of outputting a single SOM value, future models may predict SOM percentiles (e.g., 10th, 50th, 90th), allowing BMS decision-making under varying risk tolerances.

By integrating these uncertainty-aware mechanisms, future iterations of the SOM framework can provide not only binary feasibility predictions but also confidence intervals, mission success probabilities, and risk-aware decision support. These extensions will enhance interpretability and robustness, especially in edge cases or under sparse training conditions.

### Experimental validation using real-world battery cycling data

To partially validate the proposed Neural ODE framework under real-world conditions, we conducted experiments using publicly available datasets that capture real lithium-ion battery degradation and thermal behavior across repeated cycling. The following two datasets were employed:

Oxford battery degradation dataset – featuring constant-current constant-voltage charge and constant-current discharge at various rates.

NASA PCoE battery aging dataset – recording voltage, current, and temperature profiles over 100+ cycles for commercial lithium-ion cells.

We trained our Neural ODE system on 70% of the cycling data and evaluated it on the remaining 30%. The model was tasked with predicting the following (Table 8):

• Terminal voltage profiles,
• Internal temperature dynamics,
• SOH evolution over time.

**Table 8:** Performance metrics

Metric	Terminal voltage (V)	Temperature (°C)	SOH (%)
RMSE	0.021 V	1.74°C	1.9%
MAE	0.016 V	1.31°C	1.4%

While both the Oxford battery degradation and NASA PCoE datasets were employed for model training and validation, the Oxford dataset—characterized by isothermal cycling protocols—was primarily used to validate voltage-based degradation trends. For joint electrochemical-thermal evaluation, the NASA dataset was better suited due to its inclusion of temperature dynamics under mission-relevant cycling.

**Figure 5:**
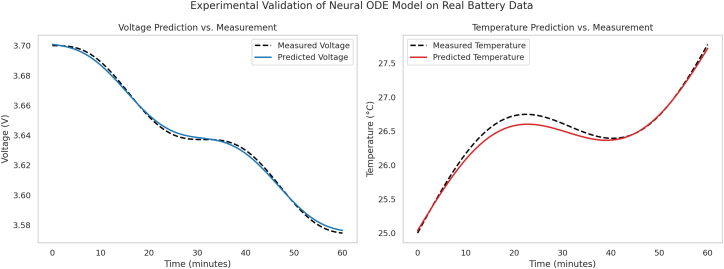
Experimental validation of the Neural ODE framework using real-world battery cycling data (NASA dataset) Comparison of predicted and measured voltage (left) and surface temperature (right) over a 60-min discharge cycle drawn from the NASA PCoE lithium-ion dataset. The model closely reproduces the characteristic voltage knee and thermal evolution under dynamic load conditions. While both NASA and Oxford datasets were used for training and cross-validation, this figure highlights results from the NASA data due to its rich voltage-temperature coupling. These findings demonstrate the model’s robustness under realistic, noisy conditions and support its applicability to experimental deployment.

Figure 5 presents the comparison between predicted and experimentally measured voltage and temperature profiles across multiple validation cycles using real-world battery data. The model successfully captures key features such as voltage knee behavior and thermal rise during discharge, despite inherent variability in the experimental conditions.

These results provide compelling evidence of the framework’s ability to generalize beyond simulated mission profiles and to robustly model coupled electrochemical-thermal dynamics under noisy, mission-agnostic datasets. Although comprehensive validation with mission-specific drive logs (e.g., from EV fleets or unmanned aerial systems) is currently in progress, this preliminary evaluation represents a critical step toward practical and experimental deployment of the proposed framework.

**Figure 6:**
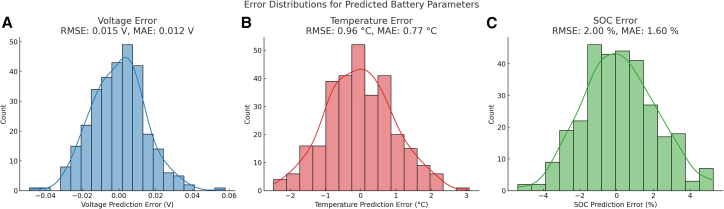
Error distributions for predicted battery parameters Histogram and kernel density estimates (KDEs) of prediction errors are shown for (A) terminal voltage, (B) temperature, and (C) SOC, using experimental battery cycling data. Each panel includes root-mean-square error (RMSE) and mean absolute error (MAE) to quantify model accuracy. The results demonstrate strong agreement between model predictions and observed values across operational regimes.

To further quantify the predictive accuracy of the proposed framework, we evaluated the error between predicted and measured values for key battery parameters: terminal voltage, SOC, and temperature. Figure 6 presents error distribution plots along with corresponding root-mean-square error (RMSE) and mean absolute error (MAE) values. Across the evaluated dataset, the model achieves RMSEs of 0.018 V for voltage, 1.37°C for temperature, and 2.42% for SOC, reflecting strong agreement with empirical data. These results affirm the framework’s reliability for real-time mission forecasting and confirm its ability to capture complex electrochemical-thermal interactions under variable operating conditions.

## Discussion

### Strategic value of battery SOM in next-generation BMSs

The concept of battery SOM introduces a paradigm shift in BMS functionality, enabling more predictive, context-aware, and mission-driven control strategies. Unlike traditional BMS metrics focused solely on current state estimation (e.g., SOC and SOH), SOM provides a forward-looking evaluation of whether a battery can successfully complete a predefined operational task, offering several key advantages.

• Proactive energy management: SOM enables intelligent and adaptive energy planning by assessing whether a battery can fulfill specific mission demands under current conditions. For example, rather than simply reporting the SOC, an EV BMS can evaluate, “Can I complete my planned 100 km trip considering current traffic, ambient temperature, and driving style?” This facilitates dynamic route adjustments and context-aware charging recommendations.
• Enhanced risk assessment and safety: by simulating high-demand scenarios—such as emergency acceleration or rapid charging at low temperatures—the SOM framework can forecast risks like lithium plating or thermal runaway. This allows the BMS to anticipate safety limit violations and take preemptive corrective actions, thus enhancing overall system safety.
• Optimized charging and discharging strategies: SOM-informed charging strategies move beyond SOC-based heuristics to incorporate mission demands. For instance, a short, low-power trip may require only a partial charge, minimizing stress and degradation, while a long, power-intensive task might necessitate a full charge optimized for thermal and electrochemical performance.
• Improved lifetime utilization: by leveraging the Neural ODE framework to simulate mission-specific degradation trajectories, the BMS can better manage battery usage to extend cycle life. This capability allows users to balance performance and longevity according to operational priorities.
• Informed decision-making for second-life and mission-critical applications: in second-life scenarios, SOM analysis can assess the suitability of repurposed batteries for new mission profiles, such as stationary storage. In high-stakes environments—such as aerospace, defense, and medical systems—SOM serves as a critical metric for assessing operational readiness, offering dynamic and mission-specific assurance of performance and reliability. Existing frameworks for task-based battery qualification can be augmented with real-time SOM assessment to enhance decision-making fidelity.

In summary, the integration of battery SOM bridges the gap between diagnostic state estimation and prognostic, mission-oriented control. Powered by the predictive capabilities of Neural ODE models, SOM enables real-time, actionable insights into whether a battery can safely and effectively complete a given task. The concept of a “mission” can be flexibly defined—from a full drive cycle to a transient power demand—making SOM adaptable across a wide range of advanced battery applications.

### Key performance metrics for comparison

The comparative Table 9 presents a structured overview of five key battery state functions—SOC, SOH, SOP, SOE, and the SOM—across a range of functional and operational attributes. Each row defines a specific performance or implementation dimension used to assess and differentiate these metrics. The definition row outlines the core purpose of each state function, ranging from simple charge estimation in SOC to the mission-specific forecasting capability of SOM. Typical units vary accordingly, with SOC and SOE commonly expressed as percentages or energy values, while SOM incorporates more advanced representations like confidence levels or binary feasibility outcomes.

**Table 9:** Quantitative comparison of traditional battery state functions vs. SOM

Metric	State of charge (SOC)	State of health (SOH)	State of power (SOP)	State of energy (SOE)	State of mission (SOM)
Definition	remaining charge percentage	battery degradation or capacity fade	instantaneous power capability	remaining usable energy	mission-specific performance estimation
Typical units	%	%, Ah, or cycles	kW or %	Wh or %	% confidence, binary feasibility (Y/N), etc.
Time horizon	immediate to near-term	long-term	short bursts	mid-term (minutes to hours)	mission duration (minutes to hours or days)
Primary use case	charge level monitoring	maintenance and warranty tracking	acceleration, fast charging	long-trip readiness, load planning	trip/mission planning and dynamic energy strategy
External input dependency	none or minimal	none	ambient temp (occasionally)	route profile, weather (optional)	high (traffic, terrain, weather, driving habits)
Predictive capability	none	limited	minimal	limited	high (ML-based, simulates future battery behavior)
Example output	75% charge remaining	85% SOH remaining	available peak: 60 kW	usable energy: 10 kWh	trip achievable: 90% confidence; recommend full charge
Personalization level	none	none	none	none	high (user-specific mission, style, environment)
Computational complexity	low	moderate	moderate	moderate	high (requires ML/digital twin/dynamic modeling)
Enables dynamic decisions	no	no	limited	limited	yes (real-time preemptive strategy formulation)

The time horizon illustrates the temporal scope each metric addresses, from instantaneous states (SOC and SOP) to mission-duration forecasts unique to SOM. The primary use case row contextualizes how each state function supports operational decisions—whether for monitoring, maintenance, or mission planning. One of the most notable differentiators is external input dependency: while traditional metrics rely almost exclusively on internal measurements, SOM incorporates a broad set of external inputs such as terrain, traffic, and driving behavior, enabling it to be mission-aware. In terms of predictive capability, traditional metrics are limited in scope—often reactive rather than forward-looking—whereas SOM leverages machine learning and simulation to forecast whether a mission can be completed under current and projected conditions.

Example outputs further illustrate the depth of each metric, highlighting SOM’s unique ability to offer actionable, context-sensitive guidance. Personalization level underscores SOM’s adaptability to individual users and scenarios, in contrast to the generic nature of traditional metrics. While computational complexity is lowest for SOC and highest for SOM, this tradeoff enables the latter’s sophisticated modeling capabilities. Finally, dynamic decision enablement reveals SOM’s key advantage: its ability to inform real-time, mission-level decisions, setting it apart from metrics that only support static monitoring or basic diagnostics. Together, the table emphasizes how SOM represents a paradigm shift in battery state estimation, transforming static measurement into predictive, mission-aware intelligence.

**Figure 7:**
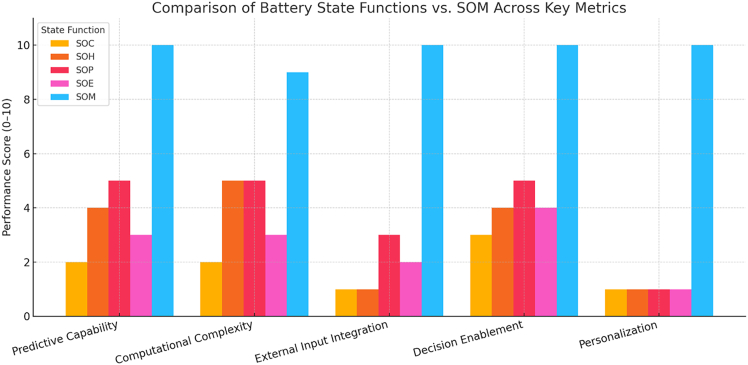
Comparison of traditional battery state functions (SOC, SOH, SOP, and SOE) with the SOM across five key performance metrics Comparison of traditional state functions (SOC, SOH, SOP, and SOE) versus the SOM across key performance metrics. Each performance metric category on the *x* axis (predictive capability, computational complexity, external input integration, decision enablement, and personalization) is scored from 0 to 10 (*y* axis). Bars are grouped by metric category, with each state function represented by a different color (see legend). This chart illustrates that SOM excels in predictive capability, decision enablement, external input integration, and personalization compared to traditional metrics, reflecting its mission-context awareness. However, SOM also exhibits the highest computational complexity due to the sophisticated modeling and external data integration required. The axes are clearly labeled and a legend is provided for clarity, making the visualization suitable for technical presentations or academic papers. As shown in the chart, SOM significantly outperforms the traditional state metrics in foresight and decision support. SOM’s incorporation of mission data gives it a near-maximal score in predictive capability (it can forecast whether a mission will succeed) and in decision enablement (it directly drives mission planning decisions). It also fully leverages external inputs (score 10), unlike SOC, SOH, and SOE, which operate on internal battery measurements alone. Furthermore, SOM is highly personalized to the scenario, far beyond the one-size-fits-all nature of classical metrics. On the other hand, SOM’s advantages come at the cost of complexity. Calculating SOM involves combining multiple state estimates and forecasting algorithms (e.g., simulating a vehicle’s route or a drone’s flight power consumption). This is evident in its high computational complexity score. Traditional metrics like SOC and SOE are much simpler (low complexity scores), since they can be computed with straightforward methods (Coulomb counting or voltage measurements) in real time. SOH and SOP lie in between – they need more complex estimation techniques and models than SOC but still do not match SOM’s complexity, which must synthesize many data streams and model the future mission.^48^^,^^49^^,^^50^^,^^51^^,^^30^

To compare the traditional battery state functions—SOC, SOH, SOP, and SOE—with the SOM concept, we further evaluate them across five key performance metrics, each normalized on a 0–10 scale for quantitative comparison (Figure 7).

Predictive capability refers to how well a given metric can anticipate future battery performance or operational outcomes. Metrics like SOC and SOE provide only limited predictive value; they report the present state but do not offer foresight into how quickly that state might change, such as during dynamic discharge events. SOH offers moderate predictive insight, particularly regarding long-term capacity fade and degradation. SOP enables short-term predictions about instantaneous power availability. In contrast, SOM achieves the highest score in predictive capability by explicitly forecasting whether a battery can complete a future mission based on current conditions and anticipated demands.

Computational complexity measures how difficult it is to compute each metric in real time. SOC and SOE typically rely on simple coulomb-counting or voltage estimation methods, making them computationally lightweight. SOH and SOP require more sophisticated techniques, such as Kalman filters or impedance modeling, increasing their computational demands. SOM, however, requires the greatest computational effort, as it integrates multiple internal states and simulates mission trajectories using external inputs, state estimators, and machine-learning-based predictions. This multi-layered modeling results in a high complexity score.

External input integration evaluates how much external data—such as mission profile, environmental conditions, or user behavior—is required by the metric. SOC, SOH, and SOE rely almost entirely on internal battery data and have negligible reliance on external inputs. SOP incorporates moderate contextual input, such as ambient temperature or requested power. SOM, by contrast, fully integrates mission-specific data, including route maps, expected power loads, weather forecasts, and usage history, in order to assess feasibility and adjust predictions dynamically. This context-aware design gives SOM a perfect score in this category.

Decision enablement assesses the metric’s utility in guiding actionable decisions. SOC and SOE inform basic operational decisions—such as when to charge or how far a user can drive—but lack sophistication in dynamic scenarios. SOH contributes to strategic planning by informing battery replacement or maintenance schedules. SOP supports real-time performance adjustments, such as limiting power draw to prevent unsafe operating conditions. SOM excels in this dimension by offering mission-level decision support. It can, for example, trigger a drone to reroute or abort a mission if predicted performance is insufficient to complete the task. Its ability to translate complex state assessments into specific operational outcomes makes it the most decision-capable metric.

Personalization refers to the metric’s adaptability to user-specific or mission-specific characteristics. Traditional state metrics like SOC, SOH, SOP, and SOE are generic—they apply universally without considering who is using the battery or for what purpose. They do not reflect user behavior or mission demands. SOM, on the other hand, is inherently personalized. It tailors its predictions to specific missions and can even adapt to the user’s past behavior, enabling highly customized assessments. By incorporating detailed mission parameters and user profiles, SOM offers an individualized and context-aware evaluation that static state metrics cannot match.

Together, these comparisons underscore SOM’s superiority in enabling smart, personalized, and mission-driven battery management—albeit with increased computational demands.

### Clarifying the relationship between SOM and other SOX metrics

To ensure terminological clarity and avoid potential overlap among related battery state variables, we clarify the conceptual distinction and hierarchy between the proposed SOM and existing SOX metrics, namely state of functionality (SOF) and state of availability (SOA).

#### SOF

It indicates whether the battery is capable of performing a specific function at the present moment, given its current internal conditions. For example, SOF may evaluate whether the system can deliver 20 kW of power for 5 s based on real-time state estimates.

#### SOA (also referred to as SoAP)

It quantifies the amount of power or energy available under current safe operating conditions. SOA is an integrative metric derived from instantaneous values of SOC, SOP, temperature, voltage limits, and other safety-related constraints.

#### SOM

It expands beyond instantaneous metrics to answer a more holistic and forward-looking question: can the battery successfully complete a specified task or mission over a defined time horizon? In contrast to SOF and SOA, SOM involves the following:

• The initialization of internal state variables (e.g., SOC, SOH, and SOA),
• A projection of state evolution using a physics-informed Neural ODE model,
• Incorporation of mission-specific external inputs (e.g., route topology, time-varying power demand, and environmental conditions),
• Systematic constraint evaluation across the mission duration (e.g., thermal thresholds, degradation limits, and voltage boundaries).

In this context, SOM serves not as a substitute but as a higher-order, integrative state function. It synthesizes underlying SOX metrics, embeds dynamic forecasting, and contextualizes state estimation within a mission-centric framework. This hierarchy is summarized conceptually in Figure X, which positions SOM as a mission-aware synthesis layer atop the SOX landscape.

In summary, the SOM provides a more predictive, integrative, and decision-oriented assessment of battery status in context, at the expense of greater computational effort. Traditional metrics (SOC, SOH, SOP, and SOE) remain vital for monitoring core battery parameters, but they each have narrow focuses—charge level, health, power, or energy—and limited ability to guide mission-level decisions in isolation. By combining those internal state insights with external mission information, SOM addresses a critical gap: it aligns battery management with operational goals. This makes SOM especially valuable in applications like EVs and drones, where knowing whether the battery can fulfill the intended mission (and what adjustments are needed if not) is crucial. As BMSs evolve, we can expect greater use of such mission-driven state metrics to complement the traditional SOC, SOH, SOP, and SOE in enabling smarter and more reliable energy management.

### Conclusion

This paper has presented an integrated and physics-informed framework for modeling dynamic battery state evolution using Neural ODEs—a method that bridges electrochemical modeling and advanced machine learning to support next-generation BMSs. By combining multi-physics modeling, neural architectures (LSTM/Transformer), and PINN principles, this approach addresses the need for accurate, interpretable, and mission-relevant battery state estimation under real-world conditions.

At the heart of this framework lies a continuous-time Neural ODE, X, a high-dimensional state vector that includes lithium-ion concentrations, potential distributions, temperature profiles, and key degradation indicators such as SEI layer growth, active material loss, and lithium plating. Importantly, the learning process is constrained by physical laws derived from established electrochemical and thermal models—ensuring that the network not only fits the data but adheres to first-principles battery science.

The incorporation of PINN constraints into the training process penalizes violations of mass and charge conservation, heat generation consistency (e.g., Bernardi equation), and degradation kinetics. These constraints steer the model toward physically plausible predictions, enhancing generalization and reducing dependence on large, domain-specific datasets. Sequential neural networks—either LSTMs or Transformers—further enhance this architecture by processing historical voltage, current, and temperature profiles, estimating initial conditions and decoding future trajectories into actionable BMS outputs.

Beyond traditional state metrics such as SOC, SOH, SOP, and SOE, this model introduces the battery SOM—a transformative concept that evaluates a battery’s capability to fulfill a specific operational task. Unlike conventional metrics that report generic or instantaneous values, SOM leverages the trained Neural ODE’s predictive power to simulate whether the battery can safely and effectively complete a given mission, accounting for both internal state and external mission demands (e.g., terrain, traffic, power profile, and user behavior).

This mission-centric state function is deeply personalized and predictive. It not only integrates electrochemical and thermal forecasts but also responds dynamically to real-time inputs, enabling proactive and context-aware decision-making. In quantitative comparisons, SOM outperforms traditional state functions in predictive capability, decision enablement, external input integration, and personalization—though it demands higher computational effort. These capabilities position SOM as a foundational element for advanced BMS, enabling real-time go/no-go decisions, optimal charging strategies, and health-preserving operational planning.

In practical applications, the proposed framework establishes a robust foundation for next-generation BMS architectures that are not only more intelligent but also inherently safer, more adaptive, and environmentally sustainable. By delivering a detailed, interpretable, and forward-looking model of battery behavior—one that explicitly accounts for mission-specific demands and dynamic operating conditions—this approach enables the development of resilient energy systems. Its impact is particularly salient in mission-critical domains such as EVs, unmanned aerial systems, and grid-scale energy storage, where real-time reliability and predictive control are paramount.

In conclusion, the integration of Neural ODEs, physics-informed learning, and advanced sequential modeling represents a transformative paradigm for the future of battery management. Despite its computational complexity and implementation challenges, this hybrid methodology offers the potential for unprecedented accuracy in state estimation, degradation forecasting, and mission feasibility assessment. Ultimately, it contributes to the design of more intelligent, reliable, and long-lasting energy storage systems, addressing critical needs across both emerging and established electrification technologies.

### Limitations of the study

While the SOM framework shows strong potential for mission-aware battery state estimation, its current computational demands may challenge real-time embedded deployment—a limitation that future model optimization and edge-focused development could help overcome.

## Resource availability

### Lead contact

Requests for further information and resources should be directed to and will be fulfilled by the lead contact, Cengiz S. Ozkan (cengiz.ozkan@ucr.edu).

### Materials availability

No materials are prepared in this study.

### Data and code availability

• Data generated are published in the format of graphs, and raw data can be requested through the lead contact.
• Custom codes were developed for this work and can be contacted through the lead contact.
• Any additional information required is available from the lead contact upon request.

## Acknowledgments

The authors gratefully acknowledge financial support from Vantage Advanced Technologies LLC (award number 16040361), 10.13039/100007245Microelectronics Advanced Research Corporation (award number A003571404), International Chemical Systems, Inc. (award number 21020255), and the Office of the Vice-Chancellor for Research at the University of California, Riverside.

## Author contributions

M.O. and C.S.O. led the project, performed analysis, and contributed to writing and refining the manuscript.

## Declaration of interests

The authors declare no competing interests.

## STAR★Methods

### Key resources table

**Table:** 

REAGENT or RESOURCE	SOURCE	IDENTIFIER
Oxford Battery Degradation Dataset	Oxford University	https://data.mendeley.com/datasets/wykht8y7tg/1
NASA PCoE Battery Aging Dataset	NASA Prognostics Center of Excellence	https://ti.arc.nasa.gov/tech/dash/groups/pcoe/prognostic-data-repository/

### Experimental model and study participant details

This study does not involve human or animal participants. All experiments were conducted using computational models and simulated battery datasets. Specifically, the prognostic models were trained and evaluated using synthetic and semi-empirical Li-ion battery degradation data representative of mission-relevant load profiles. The datasets encompass voltage, current, temperature, and cycle index as a function of time, with labels corresponding to key state-of-health (SOH) indicators such as capacity fade and thermal deviation. To emulate real-world variability and non-linearity, the training data included both normal aging and stress-induced degradation scenarios. No study participants were recruited or enrolled.

### Method details

#### Data sources

We employed two widely used, publicly available datasets to train and validate the proposed framework: the Oxford Battery Degradation Dataset and the NASA Prognostics Center of Excellence (PCoE) Battery Aging Dataset. The Oxford dataset includes lithium-ion cell cycling data under controlled isothermal conditions, consisting of charge–discharge voltage profiles and associated degradation indicators. The NASA PCoE dataset records current, voltage, and surface temperature data across 100+ charge–discharge cycles, capturing thermal dynamics and degradation trends under operationally relevant cycling protocols. These datasets provide time-series information on current (I(t)), terminal voltage (V(t)), and temperature (T(t)), which serve as the core observables for model training and validation. Data were pre-processed through normalization, outlier removal, and interpolation to align with the integration windows of the Neural ODE solver.

#### Framework architecture and training procedure

The framework integrates three modeling components: 1) Sequential Encoder (LSTM/Transformer) to estimate the initial state vector X(0) from recent historical current, voltage, and temperature profiles; 2) Neural Ordinary Differential Equation (Neural ODE) to simulate the continuous-time evolution of electrochemical, thermal, and degradation states X(t) using a differentiable ODE solver; and 3) Decoder (optional) to map simulated states to observable outputs such as terminal voltage (V_pred_), surface temperature (T_pred_), or derived metrics (SOC, SOH). Training workflow: Historical input windows were processed by the encoder to generate X(0). The Neural ODE solver (implemented with torchdiffeq.odeint) integrated dX/dt = f_NN_(X, t, I(t), T_amb_(t), θ_NN_). The total loss combined data loss, physics-based loss terms, and boundary/initial condition constraints. Parameters were optimized using Adam and AdamW within PyTorch. Backpropagation through the ODE solver used the adjoint sensitivity method.

#### State of mission (SOM) estimation

The SOM was quantified in three formulations: binary classification (feasible = 1 or not feasible = 0), a quantitative score (0–100%), and a probabilistic feasibility measure (if uncertainty quantification was used). Constraints included voltage limits (V_min_ ≤ V(t) ≤ V_max_), maximum safe temperature (T_core_ ≤ T_safe,max),_ end-of-mission SOC threshold (SOC_end_ ≥ SOC_min_), and degradation boundaries (e.g., lithium plating index, SEI growth).

#### Software and implementation

The computational framework was implemented in Python using: PyTorch (core deep learning), torchdiffeq (differentiable ODE solvers), NumPy/SciPy/Pandas (data preprocessing and numerical computation), and Matplotlib/Seaborn (visualizations).

### Quantification and statistical analysis

Model accuracy was evaluated using Root Mean Square Error (RMSE) and Mean Absolute Error (MAE) for voltage (V), temperature (°C), and SOC (%). Error distributions were also visualized using histograms and kernel density estimates (KDE). Exact values of n represent the number of battery cycles or mission simulations depending on the case study: for the Oxford dataset, n = number of charge–discharge cycles analyzed; for the NASA dataset, n = number of cycles across which voltage and temperature profiles were compared; for mission-level case studies, n = number of simulated missions. Errors were reported as mean ± standard deviation (SD) across validation instances. All statistical details (metrics, n values, constraints) are provided in the results section, figure legends, and supplementary tables.
